# Case Report: Disseminated leishmaniasis and rheumatoid arthritis: navigating a clinical conundrum

**DOI:** 10.3389/fimmu.2025.1599381

**Published:** 2025-06-05

**Authors:** Endrit Shahini, Donatello Marziliano, Francesco Losito, Marianna Zappimbulso, Elisabetta Cavalcanti, Raffaele Armentano, Fabio Fucilli, Raffaele Cozzolongo, Giuseppe Ingravallo, Fabrizio Pappagallo, Roberta Iatta, Vanessa Desantis, Annalisa Saracino, Angelo Vacca, Antonio Giovanni Solimando

**Affiliations:** ^1^ Gastroenterology Unit, National Institute of Gastroenterology-IRCCS “Saverio de Bellis”, Castellana Grotte, Bari, Italy; ^2^ Guido Baccelli Unit of Internal Medicine, Department of Precision and Regenerative Medicine and Ionian Area-(DiMePRe-J), School of Medicine, Aldo Moro University of Bari, Bari, Italy; ^3^ Histopathology Unit, National Institute of Gastroenterology, IRCCS “Saverio de Bellis” Research Hospital, Castellana Grotte, Bari, Italy; ^4^ Radiology Unit, National Institute of Gastroenterology-IRCCS “Saverio de Bellis”, Castellana Grotte, Bari, Italy; ^5^ Department of Precision and Regenerative Medicine and Ionian Area (DiMePRe-J), Pathology Unit, University of Bari “Aldo Moro”, Bari, Italy; ^6^ Interdisciplinary Department of Medicine, University of Bari “Aldo Moro”, Bari, Italy; ^7^ Pharmacology section, Department of Precision and Regenerative Medicine and Ionian Area (DiMePRe-J), University of Bari “Aldo Moro”, Bari, Italy; ^8^ Clinic of Infectious Diseases, Department of Precision and Regenerative Medicine and Ionian Area (DiMePRe-J), University of Bari “Aldo Moro”, Bari, Italy

**Keywords:** zoonosis, leishmaniasis, rheumatoid arthritis, immunosuppression, anti-CCP antibodies

## Abstract

**Background:**

Leishmaniasis is a potentially life-threatening protozoan infection that presents with many clinical manifestations, including cutaneous, mucocutaneus and visceral forms. In patients with rheumatoid arthritis (RA), cutaneous leishmaniasis can persist or re-emerge due to treatment-induced immunosuppression. However, it remains unclear whether this severe opportunistic infection is primarily driven by medication-induced immunosuppression or other poorly understood immune-mediated mechanisms that increase susceptibility.

**Case presentation:**

We describe an unusual case of disseminated leishmaniasis in a 50-year-old Italian man from Apulia, diagnosed with RA two years earlier. Following 15 months of unsuccessful immunosuppressive therapies, he developed severe multilineage pancytopenia, moderate hypertransaminasemia, elevated inflammatory markers, monoclonal gammopathy, clinically significant hepatosplenomegaly, and an ulcerated skin lesion. Initial diagnostic efforts excluded common infectious agents, primary hematological disorders, Felty syndrome, and amyloidosis. The non-specific histopathological findings from the pyoderma gangrenosum-like lesion and the transient clinical response to empirical steroids, broad-spectrum antibiotics, and granulocyte colony-stimulating factors further complicated the diagnostic process. The breakthrough came when a liver biopsy, performed to investigate persistent hypertransaminasemia, revealed *Leishmania* amastigotes within macrophages. This finding triggered a re-evaluation of the ulcerated skin lesion, and histological analysis confirmed concurrent cutaneous leishmaniasis. Subsequent bone marrow biopsy also identified *Leishmania* amastigotes, clinching the diagnosis of disseminated leishmaniasis. A holistic re-assessment of the patient’s clinical presentation, developmental history, and laboratory, radiologic, and pathological data led to the definitive diagnosis. Treatment with standard intravenous amphotericin B resulted in clinical resolution. A follow-up bone marrow biopsy a few weeks later confirmed the infection had been completely eradicated.

**Conclusions:**

In patients with rheumatological conditions, the overlapping symptoms of systemic diseases and infections like leishmaniasis can lead to significant diagnostic delays. This case underscores the importance of comprehensive and meticulous diagnostic evaluations in immunosuppressed individuals to prevent potentially fatal outcomes.

## 1 Introduction

Leishmaniasis is a potentially fatal protozoan infection with a broad spectrum of clinical manifestations, including cutaneous, mucocutaneus and visceral forms (1). The primary causative agents of visceral leishmaniasis (VL) are *Leishmania infantum* and *Leishmania donovani*, which are endemic mainly in tropical and subtropical regions worldwide. Despite extensive research, the specific risk factors for transmission remain unclear (2). *Leishmania infantum* is particularly prevalent in the Mediterranean region, including Spain, France, Italy, and Portugal (3).

Visceral leishmaniasis manifests with varying degrees of severity, ranging from acute or subacute to chronic forms, with incubation periods spanning weeks, months, or even years (1). The parasites spread via phagocytic cells within the bone marrow, spleen, liver, and lymph nodes. The most common clinical symptoms include persistent irregular fever, hepatosplenomegaly, malnutrition, pancytopenia, and hypergammaglobulinemia, predominantly characterized by elevated IgG levels due to polyclonal B cell activation (1). A distinctive feature of VL is hepatic involvement, where immune-mediated processes cause liver morphology alterations that can progress to focal fibrosis (4). In about 40% of VL cases, *Leishmania* parasites infiltrate Kupffer cells and macrophages in the liver, leading to mononuclear cell infiltration in the portal tracts and lobules. Histopathology reveals hepatocyte ballooning, terminal hepatic venule fibrosis, and pericellular fibrosis (4). Fortunately, after treatment, most patients show significant improvement in biochemical markers of liver disease.

Diagnosis of leishmaniasis relies on biopsies from infected sites—such as bone marrow, liver, and spleen—and diagnostic tools like polymerase chain reaction (PCR) of peripheral blood and serology (5). While most immunocompetent individuals recover from *Leishmania* infections due to effective macrophage activation, immunocompromised individuals are at higher risk for chronic or opportunistic VL, leading to severe and atypical outcomes (6). Conditions that impair immune responses or promote reactivation of latent *Leishmania* infections, such as solid organ or hematopoietic cell transplants, or diseases like rheumatoid arthritis (RA), hematological, and oncological conditions, are major contributors to these severe cases in non-HIV immunocompromised individuals (7). In these patients, *Leishmania* can disseminate across multiple organs simultaneously or sequentially (8), with the gastrointestinal tract, respiratory system, and liver often affected in disseminated forms (1).

In autoimmune diseases like RA, VL’s symptoms can be misinterpreted as disease progression or flare-ups, leading to diagnostic delays and worsened outcomes (7). Moreover, RA patients undergoing immunosuppressive treatments such as methotrexate, corticosteroids, disease-modifying antirheumatic drugs, or biologics are particularly vulnerable to severe leishmaniasis infections (9–17). Studies suggest that *Leishmania* parasites may persist and even resurface in RA patients after immunosuppressive treatment (18). However, it remains unclear whether this heightened susceptibility is solely due to immunosuppressive therapies or if other immune-mediated factors play a role.

Furthermore, the anti-cyclic citrullinated peptide (anti-CCP) antibody, a crucial marker in RA diagnosis and prognosis (19, 20), has been shown to yield conflicting results in distinguishing between RA and leishmaniasis. This is because diseases like tuberculosis and leishmaniasis can stimulate the production of anti-CCP, potentially correlating with disease severity and inflammation (19, 21–24).

In this report, we present a particularly complex case of leishmaniasis with simultaneous multi-organ involvement in a patient with RA. This case presents several diagnostic challenges due to overlapping clinical features and provides a comprehensive review of the current literature on this unique and rare presentation.

## 2 Case presentation

A 50-year-old man was admitted in August 2021 to the IRCCS “Saverio De Bellis” Hospital’s Gastroenterology Unit after being previously diagnosed with rheumatoid arthritis. He presented with low-grade fever, significant weight loss over a few months, scleral jaundice, and hyperchromic urine. The patient had a history of metabolic syndrome, which included insulin resistance, hyperglycemia, obesity, dyslipidemia, and hypertension. He also had non-alcoholic fatty liver disease for over 20 years and hypertransaminasemia.

The patient was diagnosed with RA within the past three years (March 2020) due to recurring symptoms in large joints and abnormal laboratory indices of inflammation associated with anti-CCP positivity. The patient was also diagnosed with latent tuberculosis and prescribed isoniazid by the rheumatologist. Following the diagnosis (March 2020), he was initially treated with methotrexate 15 mg/week for six months, but the clinical and biochemical results were not satisfactory. Therefore, Abatacept (150 mg/week) was added in September 2020 but was terminated nine months later (May 2021) due to the onset of the previously mentioned symptoms. The patient has received both doses of the SARS-COV-2 vaccination as per the recommended protocol. Moreover, the patient was treated with valacyclovir (1000 mg/day for one week) due to a weak serological positivity for HSV IgM. Additionally, he underwent surgery for the insertion of a left hip prosthesis, presumably due to osteonecrosis of the femoral head, most likely caused by the long-term use of steroids.

The patient was admitted in August 2021 to the IRCCS “Saverio De Bellis’’ Hospital due to several concerning lab signs and symptoms, including elevated bilirubin, AST, ALT, and GGT levels, along with a 10-kg weight loss over the previous three months, daytime persistent fever, and fatigue.

Upon admission, the patient’s liver function indicators were found to be up to three times higher than the normal range, including aspartate aminotransferase (AST) at 109 U/L, alanine aminotransferase (ALT) at 84 U/L, and gamma-glutamyl transferase (GGT) at 128 U/L. Other abnormalities included hyponatremia at 123 mmol/L, marked multilineage pancytopenia (Hemoglobin 9.0 g/dL, platelets at 59,000 cells per liter, leukocytes at 1,070 cells per liter, and neutrophils at 30 cells per liter), serum albumin at 2.0 g/dL, C-reactive protein (CRP) at 5.56 mg/dL, erythrocyte sedimentation rate (ESR) at 60 mm/h, ferritin at 2,859 ng/mL, procalcitonin at 0.718, beta-2 microglobulin (B2M) at 5.83, and monoclonal hypergammaglobulinemia at IgG 4 g/dL (monoclonal component, 9.6% and 11.4 g/dL) and a mild anti-smooth muscle antibody (ASMA) positivity (titer 1:80) (**Supplementary Table 1**). The serum immunofixation analysis revealed a monoclonal gammopathy with double component IgG-Kappa and IgG-lambda, while urine immunofixation was negative.

**Figure 1:**
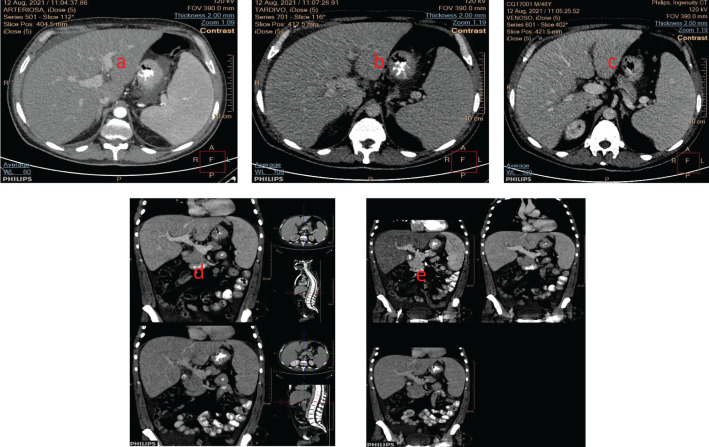
Hepatosplenomegaly was seen in the CT-scan (coronal section) in the **(a)** arterial phase; **(b)** venous phase, and **(c)** portal phase **(d)** portal (up), venous (down) phases; **(e)** arterial (left), portal (right) and venous (down) phases (sagittal sections).

Upon the initial evaluation, the patient presented with persistent fever and fatigue. Despite numerous microbiological tests, including the Widal-Wright serodiagnosis, that came back negative for Salmonella Typhi or Salmonella Paratyphi A and B infections and autoimmune tests that produced negative results, the patient required multiple blood transfusions during his hospital stay. While chest X-ray was negative, a comprehensive battery of tests, including a total body computed tomography (CT) scan, echocardiogram, and esophagogastroscopy, revealed several problems. These included hepatosplenomegaly (with the spleen diameter measuring 16 cm) (**Figure 1**), a small hepatic angioma, and enlarged subcentimetric lymph nodes in the lumbar-aortic area. Additionally, the patient displayed intra-abdominal effusion, mild hypertrophy of the left ventricle’s lateral wall with preserved global kinetics, and early esophageal varices.

A secondary cause of hepatosplenomegaly was hypothesized to be infectious or rheumatological (e.g., Felty syndrome). Furthermore, to rule out amyloidosis, a proctosigmoidoscopy with rectal biopsies was performed, with negative histopathological results.

**Figure 2:**
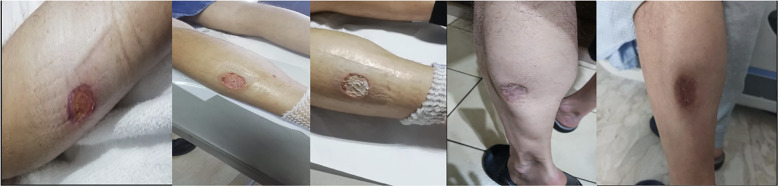
Ulcerated skin lesion on the left leg that resembles pyoderma gangrenosum, at the time of diagnosis and during the healing process.

The patient underwent surgical curettage and biopsy to treat an ulcerated skin lesion on his left leg. The lesion appeared to be pyoderma gangrenosum or cutaneous leishmaniasis (**Figure 2**), but the pathology results were initially inconclusive due to the presence of necrotic inflammatory material that hampered accurate examination.

During the first hospitalization, the patient received empiric antibiotic treatment for seven days with cefotaxime 1 gr three times daily while awaiting the results of a polymicrobial study (including blood cultures and PCR searches for herpes viruses) and autoimmune antibody tests. Then, for ten days, due to a persistent fever that was becoming less responsive to medications and exhibiting larger and more frequent peaks, piperacillin/tazobactam 4.5 gr three times daily. Only cytomegalovirus (CMV) IgG, varicella-zoster virus (VZV) IgG, and Epstein Barr virus (EBV) IgG tested positive in viral serology.

Intravenous administration of methylprednisolone was started empirically at a dose of 0.75 mg/kg/day and then gradually tapered to 40 mg/day for 5 days from August 12th to August 17th, 2021. Afterwards, the dose was decreased to 20 mg for 2 weeks from August 17th to September 3rd, followed by further tapering. Additionally, the patient was given filgrastim, a recombinant granulocyte colony-stimulating factor (G-CSF), once a week for 4 weeks. After 18 days of combined antibiotic and steroid treatment, the patient’s pancytopenia and skin leg lesion showed significant improvement (**Figure 2**), and he was finally discharged.

After a week of discharge (September 3, 2021), the prednisone dosage was gradually reduced to 12.5 mg/day, associated with only a minor improvement in blood and liver function tests over the next month.

Indeed, lab tests (October 15, 2021) revealed hemoglobin = 10.30 g/dL, platelets = 104,000 cells per liter, leukocytes = 1.310 cells per liter, neutrophils = 570 cells per liter, AST = 28 U/L, ALT = 31 U/L, GGT = 198/40 U/L, ALP = 148/129 U/L, total bilirubin = 0.54 mg/dL, C-reactive Protein = 5.70 mg/L, ESR = 102 mm/h, and albumin 2.98 g/dL, while gamma-globulins ranged from 3.24 to 3.93 g/dL (**Supplementary Table 1**).

On January 12, 2022, the patient had a rheumatological examination, which confirmed his previous diagnosis of RA and tested positive for anti-CCP. The patient’s hematological parameters and liver function tests did not improve significantly, resulting in a readmission to IRCCS “Saverio De Bellis” Hospital.

Upon admission, an abdominal ultrasound confirmed a persistently enlarged spleen (18 cm), liver and perihilar lymph node. Moreover, the most recent ultrasound revealed additional small hyperechoic formations (resembling angiomas) beyond the small subcapsular hemangioma discovered at the second hepatic segment in the previous CT scan during the first hospitalization.

Despite the patient receiving regular G-CSF treatment, there was only a slight improvement in multilineage pancytopenia with a drop in ferritin values to 522 ng/mL. Hemoglobin levels were at 10.3 g/dL, platelet counts at 115,000 cells per liter, leukocyte counts at 2,830 cells per liter, and neutrophil counts at 1,670 cells per liter. There was also some mild improvement in serum albumin values (3.0 g/dL) and liver function indicators (AST = 21 U/L, ALT = 29 U/L, GGT = 149 U). However, gamma-globulins increased further (IgG, 4.86 g/dL; monoclonal component IgG-K confirmed, 12.9%), as did inflammation markers (ESR = 93 mm/h, CRP = 1.59 mg/dL) (**Supplementary Table 1**). Among the autoimmune tests conducted, a weak positivity of ASMA was confirmed (titer 1:80). Additionally, cross-reactivity led to a false positive HBsAg that was later reversed at a second lab test, whereas HBV-DNA was negative.

**Figure 3:**
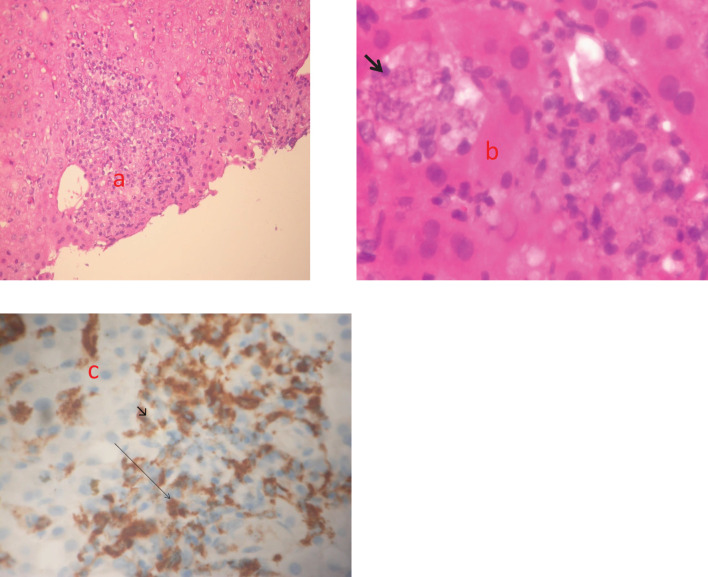
Three echotomography-assisted lunges were performed at the 7^th^ intercostal space using a semiautomatic needle (16 Gauge, 150 mm in length) to remove a whitish hepatic parenchyma frustule measuring about 10 mm; **(a)** Hepatic section stained with H&E (magnification x200). Nodular pattern of human visceral leishmaniasis (VL). Inflammatory nodule with mononuclear cells, consisting of lymphocytes, macrophages and plasma cells; **(b)** Hepatic section stained with H&E (magnification x600). Immature granulomas formed by macrophages harboring a high number of amastigotes (arrow); **(c)** Immunohistochemistry (magnification x 200). Macrophages stained with CD68 (arrowhead).

As a result, given the patient’s clinical laboratory parameters worsening, he had a liver biopsy on February 17, 2022. Pathological examination revealed unexpected evidence of *Leishmania* amastigotes inside macrophages (**Figure 3**).

**Figure 4:**
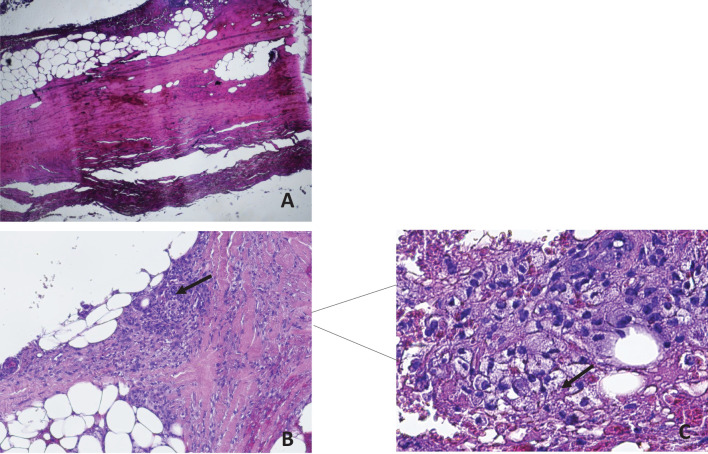
Material taken from a crusted skin lesion in the absence of the healthy structures of the epidermal lining. A 1.5 mm skin biopsy was taken from the edge of the lesions with a sterile surgical blade using 2% xylocaine as an anesthetic. The sample was fixed in 10% neutral buffered formalin (pH 7.2) for at least 78 hours, then routinely processed and sectioned at 3-4 µm and stained with hematoxylin and eosin (HE). Ulcerated and crusted parts of the skin lesion were excluded. Histological examination revealed the presence of amastigotes in the tissues colored by Giemsa, confirming the diagnosis of Leishmaniasis. **(a)** ulcerated surface of the skin with eschar and hyalurosis of the reticular dermis (HE 10X magnification); **(b)** in the subcutaneous tissue was highlighted necrotic phlogistic material with foamy histiocytes and haematoxylyphotic debris (HE 10X magnification); **(c)** HE Staining of skin biopsy revealed numerous intracellular amastigotes (4 µm) of Leishmania species (arrowheads) (HE stains, 60X magnification).

A subsequent histological revision of the ulcerated and necrotic skin lesion, prompted by a strong clinical suspicion, confirmed the diagnosis of cutaneous leishmaniasis (**Figure 4**).

The patient was admitted to the *“Guido Baccelli’’* Unit of Internal Medicine to undergo additional diagnostic workup. Laboratory results showed Haemoglobin = 10.7 g/dL, platelets = 136,000 cells per liter, leukocytes = 2.140 cells per liter, neutrophils = 1280 cells per liter, AST = 35 U/L, ALT = 51 U/L, GGT = 115 U/L, ALP = 78 U/L, total bilirubin = 0.21 mg/dL, CRP = 2.9 mg/L, ESR = 96 mm/h, albumin 2.4 g/dL, polyclonal hypergammaglobulinemia (gamma-globulins = 4 g/dL). The autoimmune panel (Antinuclear antibody, extractable nuclear antigen, anti-neutrophil cytoplasmic antibody, C3, C4, rheumatoid factor), serology for viral hepatitis, HIV, *Mycoplasma*, *Chlamydia*, and *Legionella*, as well as PCR testing for *Mycobacterium tuberculosis* on sputum and PCR for *Listeria monocytogenes*, *Streptococcus agalactiae*, *Escherichia coli*, *Neisseria meningitidis*, and *Streptococcus pneumoniae* on blood, all came back negative. Lymphocyte immunophenotyping revealed increased CD8+ and decreased CD4+ and NK cells (**Supplementary Table 1**). Also, the CT scan confirmed splenomegaly.

The clinical history and current findings indicated a suspicion of leishmaniasis, then confirmed by serology, which tested positive for IgG. It was not possible to perform IgM testing due to unavailability of the specific test. The patient had a bone marrow aspiration and biopsy (March 4, 2022). The biopsy determination revealed on histopathology hypercellular (70%) bone marrow, increased maturing components of the three blood cell lines, and dystrophic megakaryocytes. Also, several macrophages with intracellular basophilic round bodies, CD1a+, were discovered (**Supplementary Figure 1**). These findings were consistent with *Leishmania* amastigotes as confirmed by serological positivity for anti-*Leishmania* antibodies (IgG, 50.2 NTU versus 11 NTU, the upper limit normality). This was further molecularly verified testing on bone marrow aspirate by a real-time PCR targeting the 18S rRNA gene of *Leishmania* spp. (Clonit S.r.l., Milan, Italy) at the Parasitology Laboratory at the Policlinico di Bari Hospital.

The clinical manifestations and the patient’s developmental history after being re-evaluated with a holistic insight, along with all the laboratory, radiological, and histological findings, conclusively supported a diagnosis of disseminated leishmaniasis.

As a result, after being admitted to the Infectious Disease Unit, the patient’s steroid therapy was discontinued, and he was treated with liposomal amphotericin B at a dose of 3 mg/kg/day from days 1 to 5, as well as a single infusion on days 10, 17, 24, 31, and 38. He did not experience any relevant adverse events. Surprisingly, the patient fully recovered from VL, as evidenced by a new bone marrow smear and molecular analysis performed on May 11, 2022. The smear results revealed the absence of morphologically distinguishable *Leishmania* amastigotes following targeted treatment, as indicated by a negative CD1a. The hematopoietic marrow appeared slightly hypercellular (60% cellularity), with rare and small CD20+/CD3+ B/T lymphocytes in the interstitial area and two nodular aggregates. Moreover, a full recovery was suggested given the regression of monoclonal gammopathy and hypergammaglobulinemia, the gradual resolution of pancytopenia, as well as the patient’s clinical improvement immediately following treatment. Liver biopsy was proposed to the patient who refused the procedure.

This successful outcome was possible thanks to the multidisciplinary medical team’s prompt intervention and using appropriate treatment protocols, which resulted in the patient’s complete recovery from leishmaniasis. The benefit of liposomal amphotericin B, a potent antifungal agent, was especially noteworthy in this case since it is known to be more effective and less toxic than conventional amphotericin B.

Following the reintroduction of RA immunosuppressive therapy, subsequent outpatient clinical and biochemical evaluations revealed no new disease reactivation, and the patient is now fully capable of returning to his daily and work functions.

## 3 Discussion and conclusions

This case highlights the diagnostic challenges posed by leishmaniasis in patients with autoimmune diseases like RA, where overlapping clinical features can lead to misdiagnosis. The reciprocal mimicry between autoimmune and infectious diseases underscores the need for meticulous diagnostic evaluation, particularly in endemic areas or among immunosuppressed patients, to prevent delays that could result in fatal outcomes.

Symptoms such as hepatosplenomegaly, persistent fever, pancytopenia, and cutaneous lesions are shared between leishmaniasis and RA, complicating differential diagnosis (25). In our case, immunosuppressive therapy likely contributed to diagnostic delays, emphasizing the importance of heightened vigilance in similar clinical scenarios. The diagnosis of VL by *Leishmania infantum* should be suspected in RA patients, living in endemic areas including the Mediterranean regions, presenting with unexplained fever and pancytopenia. Dogs are the primary reservoir, although recent epidemiological data from areas like Taranto (Apulia) suggest that rats may contribute to the circulation of the protozoan (7).

Numerous reports link VL to RA patients on immunosuppressive or biologic therapies. Methotrexate, corticosteroids, and TNF-alpha inhibitors like adalimumab or infliximab have been associated with VL, often leading to severe outcomes (9–17, 26–35). A 2008 Greek case involved VL in a 65-year-old RA patient on methotrexate, diagnosed post-splenectomy (9), while a 2010 Spanish report linked VL to macrophage activation syndrome in a patient on adalimumab (14). Interestingly, anti-CTLA-4 therapies such as abatacept, though immunosuppressive, may modulate the immune response, reducing parasite burden and offering a potential therapeutic avenue (33–35).

Our patient, treated with abatacept for nine months, presented with pancytopenia, fever, and jaundice. Initial investigations for autoimmune flares or infectious causes, including Felty’s syndrome and leishmaniasis, were inconclusive. Liver and bone marrow biopsies ultimately confirmed VL. Treatment with liposomal amphotericin B effectively eradicated the infection, resolving both pancytopenia and monoclonal gammopathy, a rare but documented VL feature (36–39).

Autoimmune serological markers further complicate diagnosis. Anti-CCP antibodies, widely used in RA diagnosis, may be falsely elevated in infectious diseases like tuberculosis and leishmaniasis, correlating with inflammation rather than autoimmunity (19, 21–24, 40). Studies have shown significant anti-CCP levels in untreated VL patients, suggesting a possible autoimmune component triggered by parasitic infection (22, 23). This adds complexity to interpreting serological results in endemic regions for leishmaniasis.

Crucially, the patient’s path to diagnosis was significantly influenced by the comprehensive and multidisciplinary approach used. He underwent several specialized investigations, including a total body CT scan, skin lesion biopsy, liver biopsy, and bone marrow biopsy, as well as extensive blood tests. This comprehensive diagnostic strategy was critical in detecting disseminated leishmaniasis, demonstrating how thorough, targeted testing can significantly accelerate diagnosis and improve patient outcomes, especially in complex immunosuppressed cases. Accurate diagnosis of leishmaniasis requires a combination of clinical, serological, parasitological and histopathological approaches. Biopsy of affected organs, such as the liver or bone marrow, or sensitive molecular tests, such as real-time PCR on blood, remains crucial for identifying parasite-infected tissues. Prompt treatment with antiparasitic agents like liposomal amphotericin B or miltefosine is essential, along with regular follow-up to prevent relapse. Preventive measures, including insect repellent use and domestic nets, should be recommended, especially for immunocompromised patients.

In conclusion, this case underscores the need for heightened awareness and comprehensive diagnostic protocols in autoimmune patients presenting with atypical systemic symptoms. Early recognition and treatment of leishmaniasis are critical to avoid life-threatening complications, especially in populations receiving immunosuppressive therapies.

## Data Availability

The original contributions presented in the study are included in the article/**Supplementary Material**. Further inquiries can be directed to the corresponding author/s.

## Supplementary material

Supplementary Figure 1 Bone marrow biopsy highlighting hypercellular marrow with increased expression of the three maturative cell lines and dystrophic megakaryocytes. Several macrophages with round intracellular bodies compatible with leishmania amastigotes CD1a+ are within the interstitium.
