# Supplementary figures and images for: Case Report: Disseminated leishmaniasis and rheumatoid arthritis: navigating a clinical conundrum
Source: Front Immunol. 2025 Jun 5;16:1599381. doi: 10.3389/fimmu.2025.1599381 (PMC12176833; doi:10.3389/fimmu.2025.1599381)

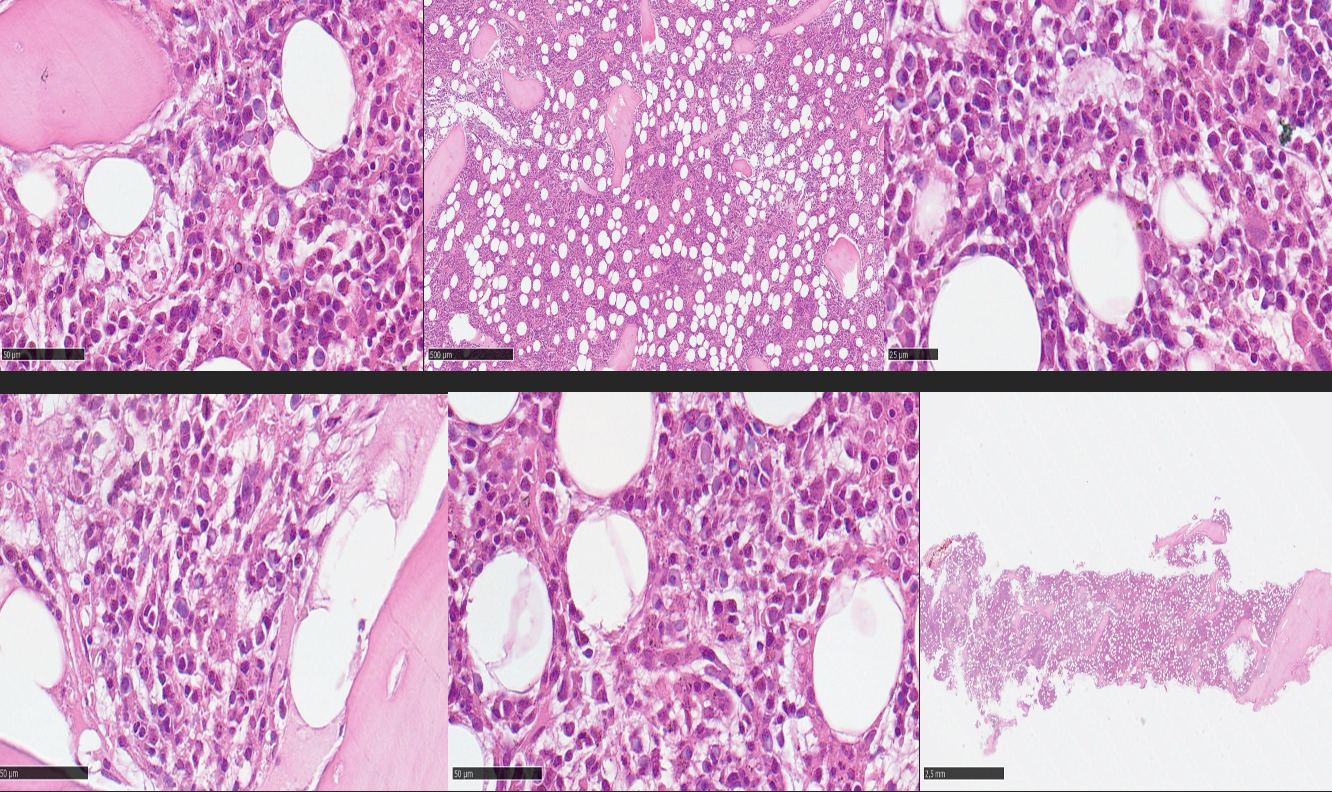

Supplement: Supplementary Figure 1 — Bone marrow biopsy highlighting hypercellular marrow with increased expression of the three maturative cell lines and dystrophic megakaryocytes. Several macrophages with round intracellular bodies compatible with leishmania amastigotes CD1a+ are within the interstitium. [file Image1.tiff]
