# Supplementary material for: Case Report: Disseminated leishmaniasis and rheumatoid arthritis: navigating a clinical conundrum
Source: Front Immunol. 2025 Jun 5;16:1599381. doi: 10.3389/fimmu.2025.1599381 (PMC12176833; doi:10.3389/fimmu.2025.1599381)
Supplement: Supplementary file 2 [file DataSheet1.pdf]

Timeline

August 2021

Admission: IRCCS ”De Bellis”

50 y.o. Male with low grade fever, jaundice, significant weight loss, leg ulcer. *Lab values* AST=109 U/L ALT=84 U/L, GGT= 128 U/L, Hb= 9 g/dL, WBCs=1,070 cell/L PLT=59000 cell/L, ferritin= 2859 ng/mL, CRP 5.56 mg/dL, ESR 60 mm/h, albumin= 2 g/dL splenomegaly (16cm diameter). *Leg ulcer biopsy: necrotic-inflammatory material*→ **Cefotaxime + Pip/Tazo+ G-CSF + steroids 0.75 mg/Kg/day.**

15th October 2021

2 months after discharge

Lab values AST=28 U/L ALT=31 U/L, GGT= 198 U/L, Hb= 10.3 g/dL, WBCs=1,310 cell/L PLT=104000 cell/L, CRP 5.7 mg/L, ESR 102 mm/h, albumin 2.98 g/dL, bilirubin= 0.54 mg/dL.

12th January 2022

Readmission: IRCCS “De Bellis”

Lab values AST=21 U/L ALT=29 U/L, GGT= 149 U/L, Hb= 10.3 g/dL, WBCs=2,830 cell/L PLT=115000 cell/L, CRP 1.59 mg/dL, ESR 93 mm/h, splenomegaly (18cm). *Liver biopsy*: Macrophages rich in amastigotes. *Leg ulcer biopsy revision:* foamy histiocytes with amastigotes.

15th February 2022

“G.Baccelli” Internal Medicine Unit

Lab values AST=35 U/L ALT=51 U/L, GGT= 115 U/L, Hb= 10.7 g/dL, WBCs=2,140 cell/L PLT=136000 cell/L, CRP 2.9 mg/L, ESR 96 mm/h, albumin 2.4 g/dL, bilirubin= 0.21 mg/dL.

4th March 2022

BM biopsy

Macrophages with intracellular basophilic round CD1a+ bodies→ **Amphotericin B 3 mg/Kg/day** → following BM biopsy CD1a-
